# iPSCs ameliorate hypoxia-induced autophagy and atrophy in C2C12 myotubes via the AMPK/ULK1 pathway

**DOI:** 10.1186/s40659-023-00435-4

**Published:** 2023-06-03

**Authors:** Haimei Cen, Pin Fan, Yuting Ding, Bin Luo, Hong Luo, Menglong Chen, Yu Zhang

**Affiliations:** 1 Department of Neurology, The First Affiliated Hospital, Jinan University, Guangzhou, 510630 Guangdong China; 2 Department of Neurology, Meizhou People’s Hospital, Meizhou, 514000 Guangdong China

**Keywords:** Duchenne muscular dystrophy, Hypoxia, Co-culture, LC3II/LC3I, Atrogin-1, MuRF-1, AMPK/ULK1 pathway

## Abstract

**Background:**

Duchenne muscular dystrophy (DMD) is an X-linked lethal genetic disorder for which there is no effective treatment. Previous studies have shown that stem cell transplantation into mdx mice can promote muscle regeneration and improve muscle function, however, the specific molecular mechanisms remain unclear. DMD suffers varying degrees of hypoxic damage during disease progression. This study aimed to investigate whether induced pluripotent stem cells (iPSCs) have protective effects against hypoxia-induced skeletal muscle injury.

**Results:**

In this study, we co-cultured iPSCs with C2C12 myoblasts using a Transwell nested system and placed them in a DG250 anaerobic workstation for oxygen deprivation for 24 h. We found that iPSCs reduced the levels of lactate dehydrogenase and reactive oxygen species and downregulated the mRNA and protein levels of BAX/BCL2 and LC3II/LC3I in hypoxia-induced C2C12 myoblasts. Meanwhile, iPSCs decreased the mRNA and protein levels of atrogin-1 and MuRF-1 and increased myotube width. Furthermore, iPSCs downregulated the phosphorylation of AMPKα and ULK1 in C2C12 myotubes exposed to hypoxic damage.

**Conclusions:**

Our study showed that iPSCs enhanced the resistance of C2C12 myoblasts to hypoxia and inhibited apoptosis and autophagy in the presence of oxidative stress. Further, iPSCs improved hypoxia-induced autophagy and atrophy of C2C12 myotubes through the AMPK/ULK1 pathway. This study may provide a new theoretical basis for the treatment of muscular dystrophy in stem cells.

**Supplementary Information:**

The online version contains supplementary material available at 10.1186/s40659-023-00435-4.

## Background

Duchenne muscular dystrophy (DMD) is a lethal muscle disease caused by spontaneous mutations in the dystrophin gene, located on chromosome Xp21.2. Hypoxemia due to respiratory dysfunction is a common late clinical manifestation of DMD and is generally thought to be the result of progressive skeletal muscle atrophy involving respiratory muscles. However, many studies have shown that localized hypoxia and oxidative damage occur in the skeletal muscles of patients with early DMD or mdx mice [1, 2]. This may be related to the fact that DMD causes defects in vascular function and angiogenesis localized in muscle tissue. It has been shown that in DMD, dystrophin deficiency leads to mislocalization of neuronal nitric oxide synthase(nNOS), which results in reduced paracrine signals from muscle-generated nitric oxide (NO) to the microvascular system, making muscle fibers more susceptible to functional ischemia during exercise [3, 4]. In addition, capillary density is reduced in muscular dystrophies, which may be related to the reduced expression of hypoxia-induced factor (HIF-1α) and vascular endothelial growth factor (VEGF) in satellite cells of dystrophic muscle, both of which can lead to reduced blood flow to the muscle tissue and insufficient oxygen supply [5, 6].

Induced pluripotent stem cells (iPSCs) are mature somatic cells of humans or animals that have been induced to reprogram pluripotent stem cells with self-renewal ability and multi-directional differentiation potential [7]. Currently, the aim of cell replacement therapy in DMD is to import new cells into patients to restore dystrophin production. There are increasingly studies in stem cell transplantation for DMD [8–11]. The transplantation of iPSC-derived cells from patients does not induce the immune response observed in allogeneic transplants; therefore, iPSCs have been identified as an important source for cell therapy for DMD [12]. Despite restoring the expression of dystrophin proteins and improving skeletal muscle function in different models, iPSCs transplantation for DMD is still far from the clinical application stage, emphasizing the need for more in-depth studies prior to clinical translation. Moreover, there are no reported studies on iPSCs transplantation to improve muscle autophagy and atrophy.

The skeletal muscle is a major protein store and is one of the most metabolically active tissues in the body. Skeletal muscle mass is determined by the balance between rates of protein synthesis and degradation. When the rate of muscle protein degradation is greater than that of synthesis, skeletal muscle mass is lost, which is often referred to as muscle atrophy or wasting. Two of the more widely studied protein degradation pathways are the ubiquitin–proteasome system (UPS) and the autophagy-lysosome pathway (ALP). The UPS is one of the major protein hydrolysis mechanisms in skeletal muscle, including the well-known muscle-specific E3 ubiquitin ligases, muscle RING finger1 (MuRF1) and muscle atrophy F-box (MAFbx, atrogin-1) [13–15]. ALP is another classical catabolic mechanism by which cells initiate a lysosome-dependent self-digestion process to maintain essential cellular activities under stressful conditions such as nutrient deprivation and oxidative damage [16]. Studies suggest that the inhibition of autophagy may be detrimental to skeletal muscle and could lead to myofiber degeneration and weakness in muscle disorders [17, 18]. However, excessive activation of the autophagy-lysosome pathway, causing massive breakdown of muscle proteins, is also responsible for muscle atrophy [19, 20]. Therefore, it is crucial to further explore the interrelationship between muscle atrophy and autophagy, not only to help study the pathogenesis of muscle atrophy but also to provide guidance on the use of drugs, treatment, and prevention.

Hypoxia is an important pathophysiological condition that induces a plethora of cellular adaptive responses and is usually accompanied by energy metabolic depletion and nutritional deficiencies. AMP-activated protein kinase (AMPK) is an energy sensor that is activated under conditions of energy deficiency. Various tissues and cells have been reported to activate AMPK to produce ATP and to restore energy homeostasis under hypoxic conditions through different molecular mechanisms [21]. Further, a recent study showed that AMPK can initiate autophagy by regulating Unc-51 like in autophagy activating kinase 1 (ULK1) [22, 23]

To determine whether iPSCs transplantation has a protective effect against hypoxic injury to skeletal muscle in DMD, we constructed a co-culture model of iPSCs and C2C12 myoblasts. In this study, we found that the iPSCs co-culture enhanced the resistance of C2C12 myoblasts to hypoxic injury and improved the autophagy and atrophy of C2C12 myotubes under hypoxia. Further, the protective effect on hypoxia-induced injury of C2C12 myotubes might be achieved through the AMPK/ULK1 pathway.

## Results

### Identification of iPSCs

**Fig. 1:**
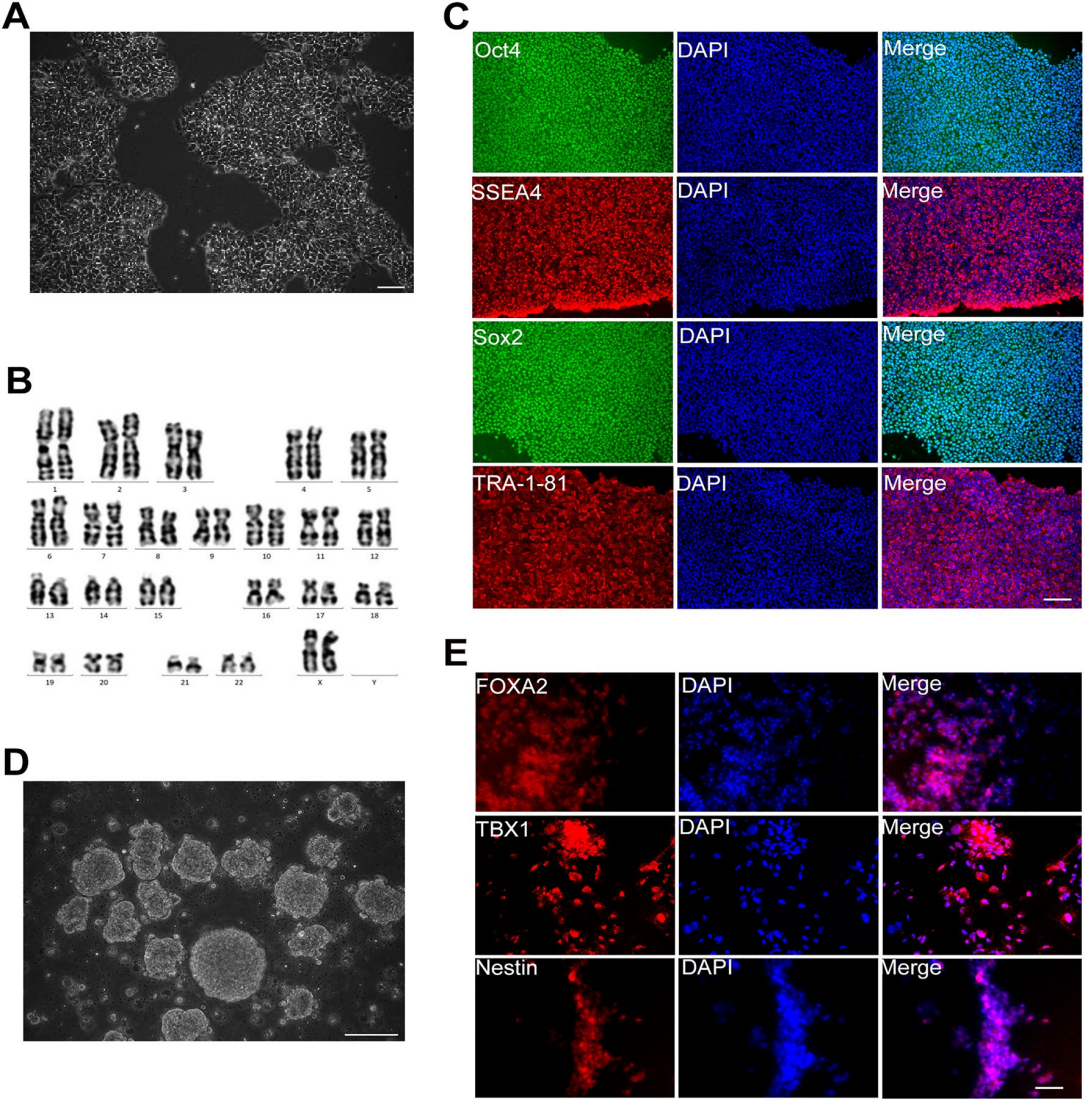
Identification of iPSCs. **A** Growth of iPSCs in serum-free, feeder layer-free cell culture medium under phase contrast microscopy. **B** Karyotype analysis of iPSCs. **C** Immunofluorescence staining showing that iPSCs expressed pluripotent stem cell markers Oct4, SSEA4, Sox2, TRA-1-81. **D** iPSCs grew as spherical embryos during spontaneous differentiation in suspension culture under phase contrast microscopy. **E** Immunofluorescence staining showing that iPSCs spontaneously differentiated into three germ layers, with FOXA2 in the endoderm, TBX1 in the mesoderm, and Nestin in the ectoderm. Scale bar = 100 μm

The normal human derived iPSCs grew in multicellular colonies with clear, smooth colony edges and fusible growth between colonies (Fig. 1A). The results of karyotype analysis showed that there were not chromosome number or structural abnormalities (Fig. 1B). Immunofluorescence staining results showed that iPSCs highly expressed pluripotent stem cell markers, such as Oct4, SSEA4, Sox2, and TRA-1–81 (Fig. 1C). Meanwhile, iPSCs spontaneously differentiated into three germ layers and grew as spherical embryos during spontaneous differentiation in a suspension culture (Fig. 1D). Immunofluorescence staining results showed that iPSCs expressed a three-germ layer marker, such as the endoderm marker FOXA2, mesoderm marker TBX1, and ectoderm marker Nestin (Fig. 1E).

### iPSCs co-culture model construction

**Fig. 2:**
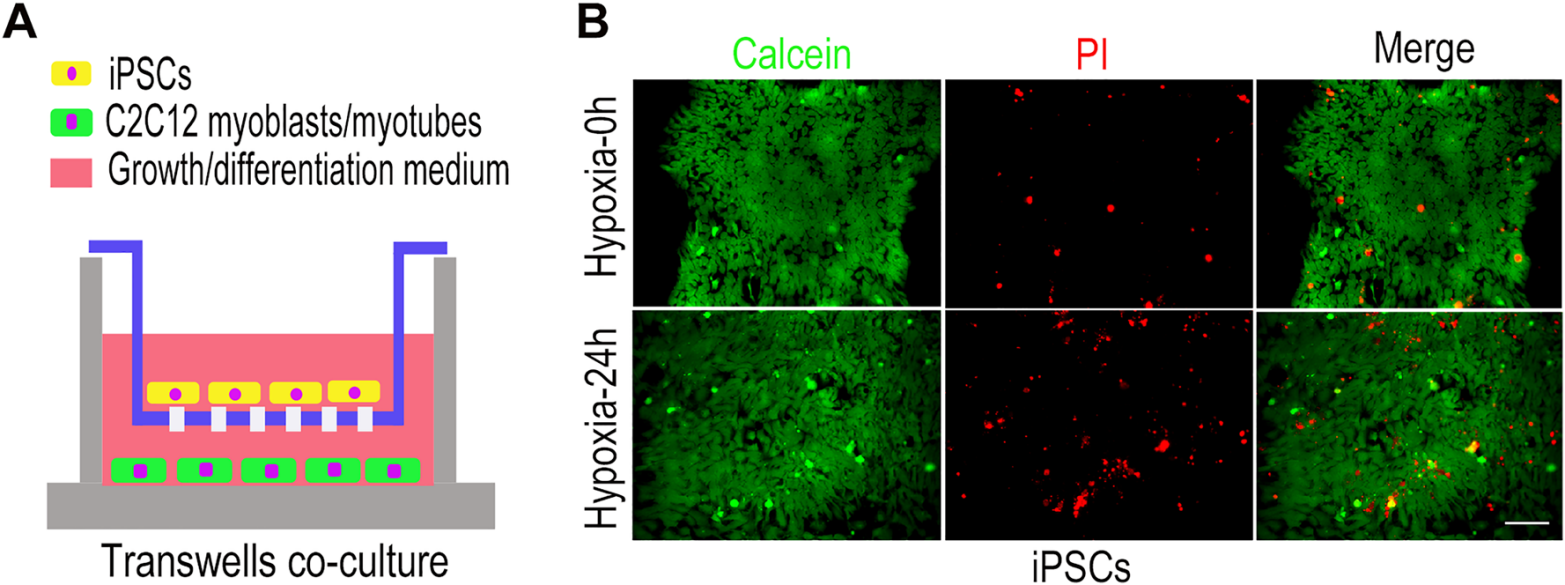
iPSCs co-culture model construction. **A** Transwell co-culture model with iPSCs cultured in a transwell insert, and C2C12 myoblasts cultured in a 6-well plate with the growth or differentiation medium of C2C12 myoblasts. **B** Cellular activity of iPSCs in C2C12 myoblasts growth medium before and after exposure to hypoxia for 24 h, where Calcein (green) indicates live cells and PI (red) indicates dead cells. Statistics were from three independent replicates of the samples and data were expressed as mean ± SEM; the remaining two groups were compared with the hypoxic group, respectively, and *P* values are the results of one-way ANOVA. *P < 0.05, **P < 0.01, ***P < 0.001. ns, no significant difference. Scale bar = 100 μm. PI: Propidium iodide

To investigate whether iPSCs protect skeletal muscles from hypoxic injury, we constructed a co-culture model of iPSCs and C2C12 myoblasts. The Transwell nested system can help us construct this co-culture model by culturing iPSCs in a Transwell insert and C2C12 myoblasts in a 6-well plate, and the upper and lower fluids were connected (Fig. 2A). To ensure that iPSCs functioned properly under co-culture conditions, we used growth medium of C2C12 myoblasts to culture iPSCs after exposure to hypoxia for 24 h. The results showed that the majority of iPSCs remained active after 24 h of hypoxic treatment (Fig. 2B).

### iPSCs enhanced the activity of C2C12 myoblasts under hypoxic damage

**Fig. 3:**
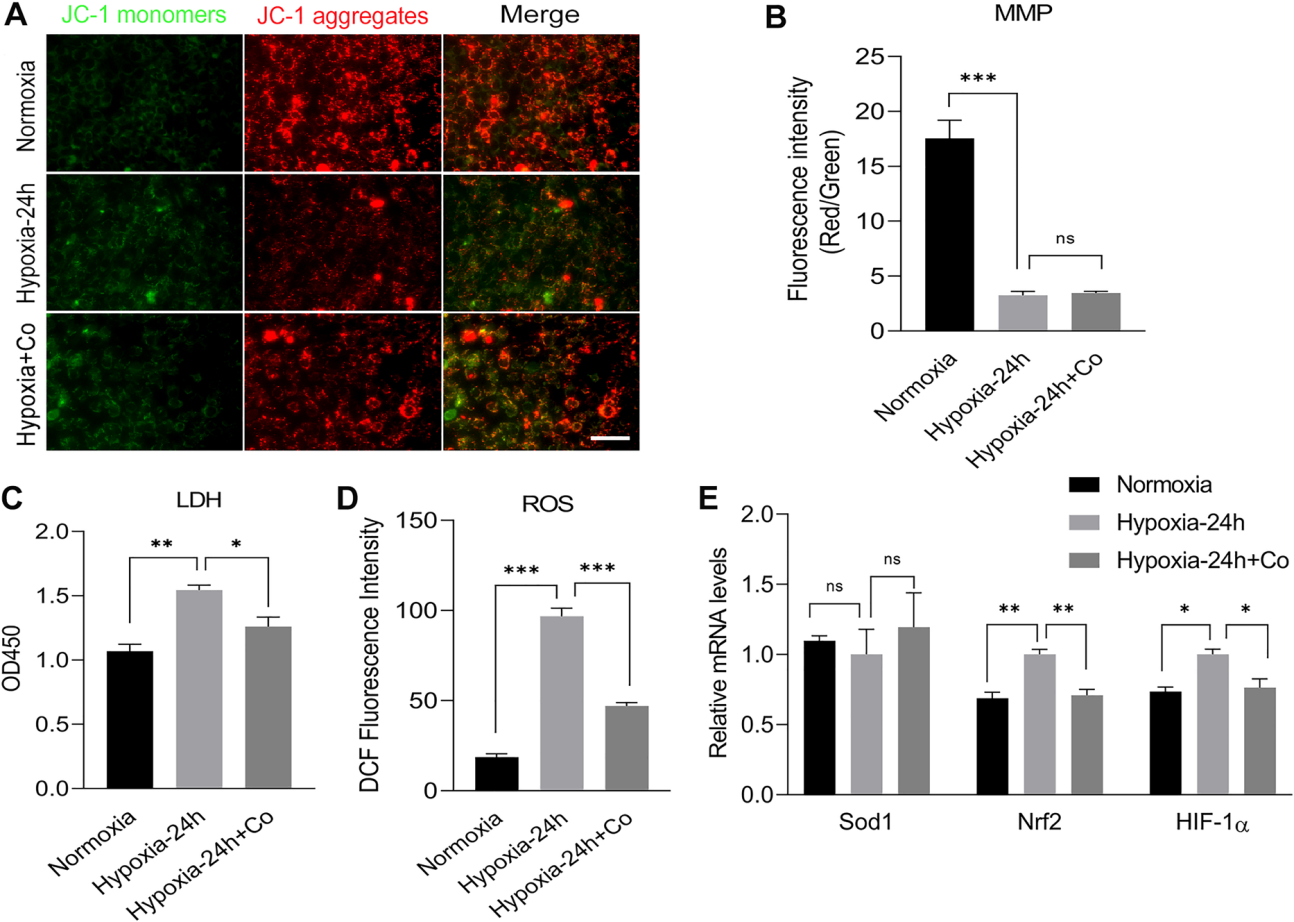
iPSCs enhanced the activity of C2C12 myoblasts under hypoxic damage. **A**, **B** Immunofluorescence staining showing the mitochondrial membrane potential of C2C12 myoblasts and the mean fluorescence intensity integral ratio of red–green fluorescence of mitochondrial membrane potential of C2C12 myoblasts after exposure to normoxia or hypoxia for 24 h with or without iPSCs co-culture. **C** Absorbance of LDH measured at 450 nm in C2C12 myoblasts medium supernatants after exposure to normoxia or hypoxia for 24 h with or without iPSCs co-culture. **D** Average fluorescence intensity integral values of ROS of C2C12 myoblasts after exposure to normoxia or hypoxia for 24 h with or without iPSCs co-culture. **E** Results of qPCR analysis showing the mRNA levels of Sod2, Nrf2, and HIF1 after after exposure to normoxia or hypoxia for 24 h with or without iPSCs co-culture. Statistics were from three independent replicates of the samples and data were expressed as mean ± SEM; the remaining two groups were compared with the hypoxic group, respectively, and *P* values are the results of one-way ANOVA. *P < 0.05, **P < 0.01, ***P < 0.001. ns, no significant difference. Scale bar = 100 μm. Co: iPSCs and C2C12 co-culture. MMP: Mitochondrial membrane potential. LDH: Lactate dehydrogenase. ROS: Reactive oxygen species

The cell viability of C2C12 myoblasts was determined when the iPSCs and C2C12 myoblasts were co-cultured in a DG250 anaerobic workstation for oxygen deprivation for 24 h. The results showed that compared to normoxic control group, hypoxia reduced the mitochondrial membrane potential in C2C12 myoblasts, and the iPSCs had no effect on this change (Fig. 3A, B). Hypoxia increased the level of LDH release from C2C12 myoblasts compared to normoxic control group, and the iPSCs reversed this change (Fig. 3C). Similarly, compared to normoxic control group, hypoxia caused an increase in ROS levels in C2C12 myoblasts and iPSCs reduced ROS levels (Fig. 3D). qPCR analysis showed that hypoxia increased the mRNA levels of Nrf2 and HIF1 in C2C12 myoblasts compared to normoxic control group, and iPSCs reversed this change (Fig. 3E). These results suggest that iPSCs enhanced C2C12 myoblasts viability under hypoxia and enhanced C2C12 myoblasts resistance against oxidative stress injury.

### iPSCs had no effect on proliferation of C2C12 myoblasts under hypoxic damage

**Fig. 4:**
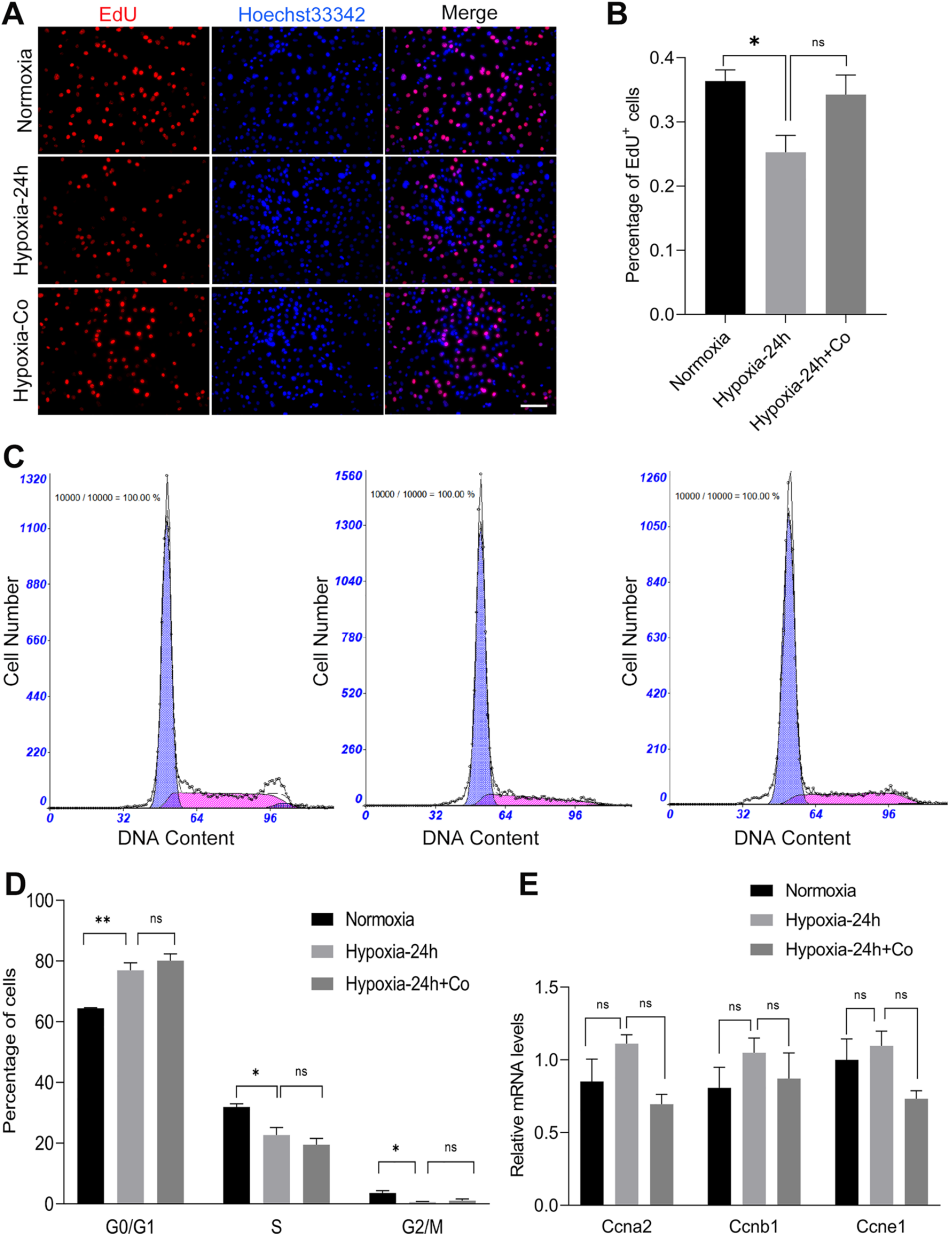
iPSCs had no effect on proliferation of C2C12 myoblasts under hypoxic damage. **A** Immunofluorescence staining showing EdU^+^ cells in C2C12 myoblasts after exposure to normoxia or hypoxia for 24 h with or without iPSCs co-culture. (red, EdU; blue, Hoechst33342). **B** The percentage of EdU^+^ cells in C2C12 myoblasts. **C** The flow chart of C2C12 DNA content. **D** The percentage of C2C12 myoblasts in each part of the cell cycle in after exposure to normoxia or hypoxia for 24 h with or without iPSCs co-culture. **E** Results of qPCR analysis showing that the mRNA levels of Ccna2, Ccnb1, and Ccne1 had no effect on C2C12 myoblasts after exposure to normoxia or hypoxia for 24 h with or without iPSCs co-culture. Statistics were from three independent replicates of the samples and data were expressed as mean ± SEM; the remaining two groups were compared with the hypoxic group, respectively, and *P* values are the results of one-way ANOVA. *P < 0.05, **P < 0.01, ***P < 0.001. ns, no significant difference. Scale bar = 100 μm. Co: iPSCs and C2C12 co-culture. EdU: 5-Ethynyl-20-deoxyuridine

To further study whether iPSCs increased the activity of C2C12 cells by affecting cell proliferation, we examined the proliferation and cell cycle of C2C12 myoblasts. In our study, immunofluorescence staining showed that after exposure to hypoxia for 24 h, the percentage of EdU-positive cells was reduced compared with that in the normoxia group. Further, iPSCs increased the percentage of EdU-positive cells, but the difference was not statistically significant (Fig. 4A, B). The DNA content of C2C12 myoblasts was measured by flow cytometry, and the analysis showed that the percentage of cells in the G0/G1 phase increased and the percentage of cells in the S phase decreased in the hypoxic and iPSC co-culture groups compared to those in the normoxic control group (Fig. 4C, D). Similarly, qPCR analysis showed that the mRNA levels of Ccna2, Ccnb1, and Ccne1 did no effect on C2C12 myoblasts between the three groups (Fig. 4E). These results indicate that the proliferation of C2C12 myoblasts was inhibited after exposure to hypoxia for 24 h, and iPSCs did not improve this change.

### iPSCs reduced the autophagy and apoptosis of C2C12 myoblasts under hypoxia

**Fig. 5:**
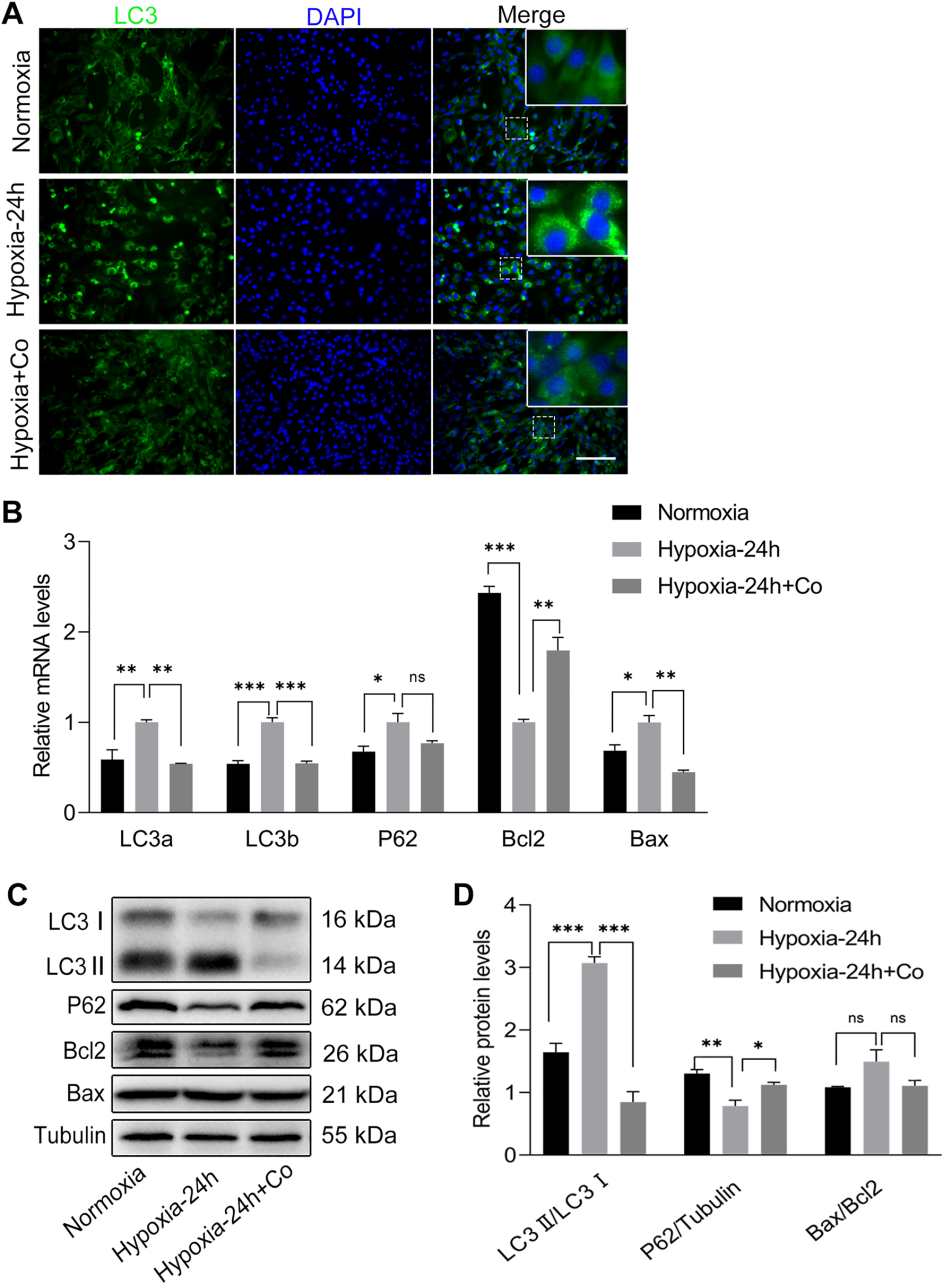
iPSCs reduced the autophagy and apoptosis in C2C12 myoblasts under hypoxia. **A** Immunofluorescence staining showing that after exposure to hypoxia for 24 h, LC3 accumulated in C2C12 myoblast perinuclear area, while in the normoxia and iPSCs co-culture groups, LC3 was diffusely distributed in the cytoplasm, (green, LC3; blue, DAPI). **B** Results of qPCR analysis showing the mRNA levels of autophagy- and apoptosis-related markers in C2C12 myoblasts after exposure to normoxia or hypoxia for 24 h with or without iPSCs co-culture. **C**, **D** Western blot results showing the protein levels of autophagy- and apoptosis-related markers in C2C12 myoblasts after exposure to normoxia or hypoxia for 24 h with or without iPSCs co-culture. Statistics were from three independent replicates of the samples and data were expressed as mean ± SEM; the remaining two groups were compared with the hypoxic group, respectively, and *P* values are the results of one-way ANOVA. *P < 0.05, **P < 0.01, ***P < 0.001. ns, no significant difference. Scale bar = 100 μm. Co: iPSCs and C2C12 co-culture

Since autophagy and apoptosis are usually activated when cells are under hypoxia, we examined the levels of autophagy- and apoptosis-related markers in C2C12 myoblasts under normoxic and hypoxic conditions for 24 h, with or without iPSC co-culture. Immunofluorescence staining showed that after exposure to hypoxia for 24 h, LC3 accumulated in the C2C12 perinuclear area, whereas in the normoxia and iPSC co-culture groups, LC3 was diffusely distributed in the cytoplasm. This indicates that hypoxia increased the autophagy level of C2C12 myoblasts, whereas iPSCs reduced the hypoxia-induced changes in C2C12 myoblasts (Fig. 5A). This is consistent with the results of qPCR and western blot analysis, which showed that iPSCs downregulated the mRNA expression of LC3a, LC3b, and P62 in C2C12 myoblasts, which were upregulated under hypoxia (Fig. 5B). Meanwhile, iPSCs downregulated the protein levels of LC3II/LC3I and increased the protein levels of P62, which were upregulated under hypoxia (Fig. 5C, D). In addition, qPCR analysis showed that hypoxia increased the expression of Bax and decreased the expression of Bcl2 in C2C12 myoblasts, and the iPSCs was able to reverse this change (Fig. 5B), but there was no statistically significant difference in the protein expression of Bax/Bcl2 between the three groups (Fig. 5C, D).

### iPSCs improved the autophagy and atrophy of C2C12 myotubes under hypoxia

**Fig. 6:**
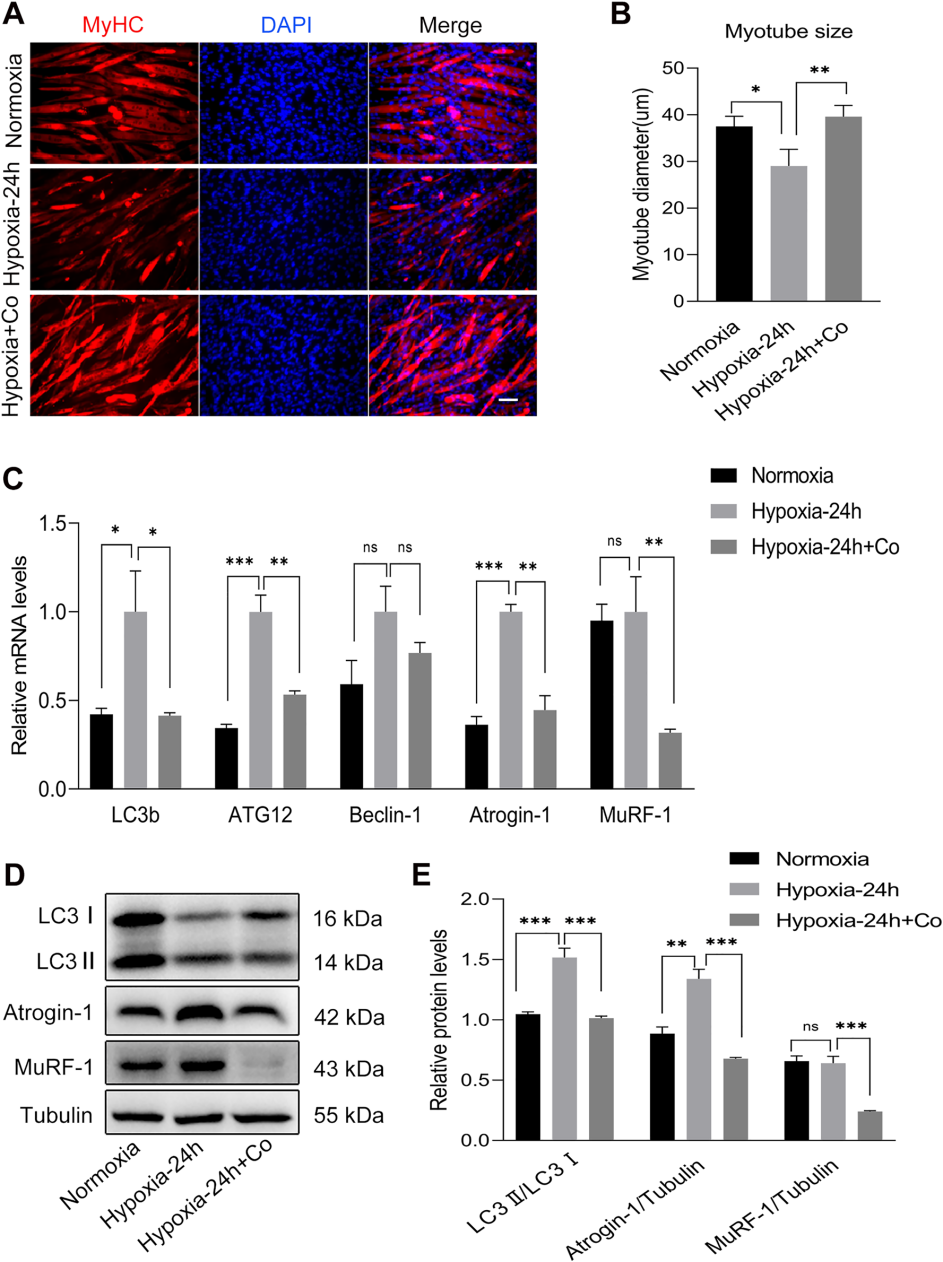
iPSCs improved the autophagy and atrophy in C2C12 myotubes under hypoxia. **A** Immunofluorescence staining showing MyHC expression in C2C12 myotubes after exposure to normoxia or hypoxia for 24 h with or without iPSCs co-culture. (red, MyHC; blue, DAPI). **B** Statistical analysis of the maximum diameter of MyHC-positive myotubes. **C** qPCR analysis results showing the mRNA levels of autophagy- and atrophy-related markers in C2C12 myotubes after exposure to normoxia or hypoxia for 24 h with or without iPSCs co-culture. **D**, **E** Western blot results showing the protein levels of autophagy- and atrophy-related markers in C2C12 myotubes after exposure to normoxia or hypoxia for 24 h with or without iPSCs co-culture. Statistics were from three independent replicates of the samples and data were expressed as mean ± SEM; the remaining two groups were compared with the hypoxic group, respectively, and *P* values are the results of one-way ANOVA. *P < 0.05, **P < 0.01, ***P < 0.001. ns, no significant difference. Scale bar = 100 μm. Co: iPSCs and C2C12 co-culture

In addition to causing skeletal muscle death, hypoxia often leads to decreased muscle mass and skeletal muscle atrophy. Autophagy is considered a key player in muscle protein degradation. Therefore, we further verified whether iPSCs could improve the autophagy and atrophy of C2C12 myotubes under hypoxic condition. We co-cultured iPSCs with C2C12 myotubes which were induced differentiation on the 5th day using a Transwell nested system and exposed them to a DG250 anaerobic workstation for oxygen deprivation for 24 h to detect C2C12 myotube autophagy- and atrophy-related markers. Immunofluorescence staining showed that hypoxia caused a decrease in the diameter of C2C12 myotubes compared to the normoxic control group, whereas iPSCs increased the diameter of myotubes compared to the hypoxic group, with no less than 90 myotubes per group at the time of counting (Fig. 6A, B). qPCR analysis results showed that the iPSCs decreased the mRNA levels of LC3b, ATG12, atrogin-1, and MuRF-1 in C2C12 myotubes, which were upregulated under hypoxia (Fig. 6C). Western blot results showed that hypoxia increased the protein levels of LC3II/LC3I and atrogin-1 in C2C12 myotubes, whereas iPSCs decreased the protein levels of LC3II/LC3I, atrogin-1, and MuRF-1 in C2C12 myotubes (Fig. 6D, E), indicating that iPSCs improved the autophagy and atrophy of C2C12 myotubes under hypoxia and protect C2C12 myotubes from oxidative damage.

### AMPK/mTOR/ULK1 pathway play an important role in the autophagy and atrophy of C2C12 myotubes under hypoxia

**Fig. 7:**
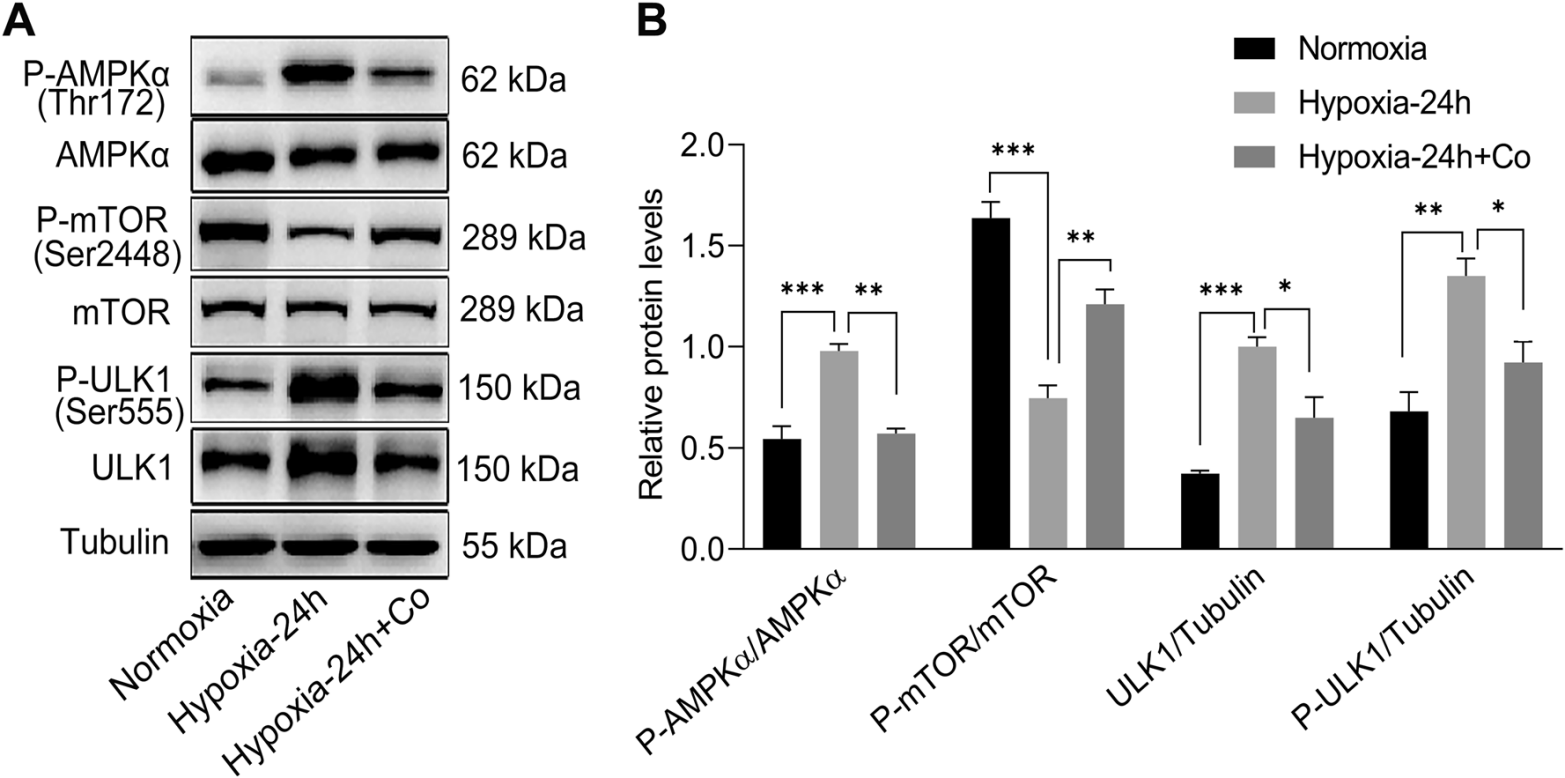
AMPK/mTOR/ULK1 pathway is involved in the autophagy and atrophy of C2C12 myotubes under hypoxia. **A**, **B** Western blot results showing AMPKα, ULK1, and mTOR total protein and phosphorylation in C2C12 myotubes after exposure to normoxia or hypoxia for 24 h with or without iPSCs co-culture. Statistics were from three independent replicates of the samples and data were expressed as mean ± SEM; the remaining two groups were compared with the hypoxic group, respectively, and *P* values are the results of one-way ANOVA. *P < 0.05, **P < 0.01, ***P < 0.001. ns, no significant difference. Co: iPSCs and C2C12 co-culture

To further investigate the protective mechanism that iPSCs improved the autophagy and atrophy of C2C12 myotubes under hypoxia, we examined signaling pathway proteins that are closely associated with autophagy initiation. Western blotting results showed that after exposure to hypoxia for 24 h, the phosphorylation of AMPKα and ULK1 were increased and the phosphorylation of mTOR were decreased in C2C12 myotubes, compared to normoxia control group (Fig. 7A, B), indicating that hypoxia activated the AMPK/mTOR/ULK1 pathway in C2C12 myotubes. Interestingly, iPSCs reversed this change, that is, the phosphorylation of AMPKα and ULK1 were downregulated, and the phosphorylation of mTOR were upregulated in C2C12 myotubes in the iPSC co-culture group after exposure to hypoxia for 24 h (Fig. 7A, B). Therefore, iPSCs may protect C2C12 myotubes against hypoxia-induced autophagy and atrophy by inhibiting the AMPK/mTOR/ULK1 pathway.

### AMPK and ULK1 inhibitors alleviated the autophagy and atrophy of C2C12 myotubes under hypoxia

**Fig. 8:**
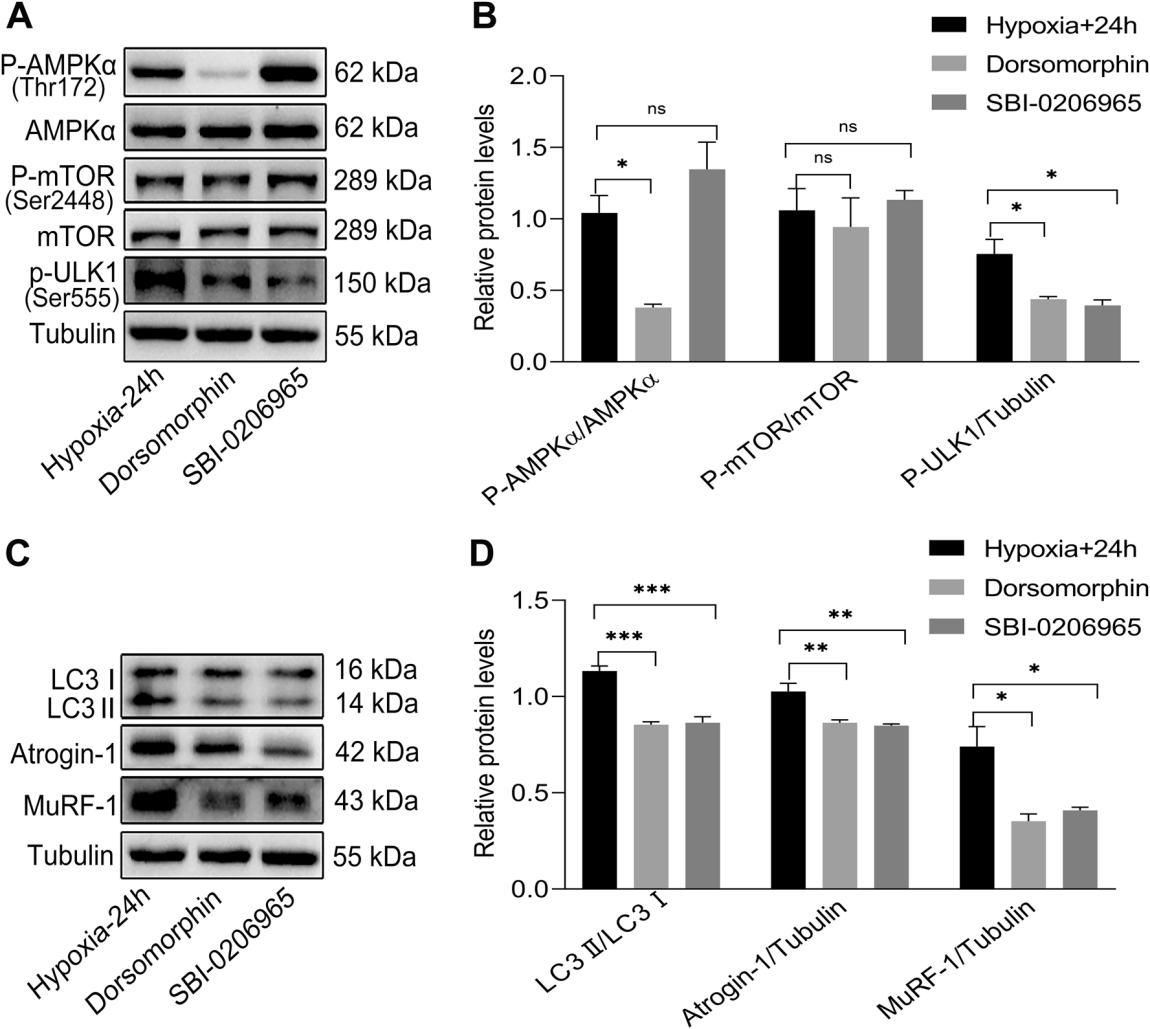
AMPK and ULK1 inhibitors alleviated the autophagy and atrophy of C2C12 myotubes under hypoxia. **A**–**D** Western blot results showing total and phosphorylation of AMPKα, ULK1, and mTOR and autophagy- and atrophy-related protein levels in C2C12 myotubes with and without AMPK inhibitor, Dorsomorphin, and ULK1 inhibitor, SBI-0206965, after exposure to hypoxia for 24 h. Statistics were from three independent replicates of the samples and data were expressed as mean ± SEM; the remaining two groups were compared with the hypoxic group, respectively, and *P* values are the results of one-way ANOVA. *P < 0.05, **P < 0.01, ***P < 0.001. ns, no significant difference. Co: iPSCs and C2C12 co-culture

To further verify whether the AMPK/mTOR/ULK1 pathway is involved in regulating the autophagy and atrophy in C2C12 myotubes under hypoxia, we added the AMPK inhibitor Dorsomorphin (0.5 μM) and ULK1 inhibitor SBI-0206965 (0.5 μM), respectively, to culture C2C12 myotubes on the 5th day of differentiation for 24 h. Protein levels of the AMPK/mTOR/ULK1 pathway and autophagy- and atrophy-related markers were detected by western blotting. The results showed that, compared to the hypoxic control group, Dorsomorphin inhibited the phosphorylation of AMPKα and ULK1 and decreased the protein levels of LC3II/LC3I, Atrogin-1, and MuRF-1 in C2C12 myotubes, but had no effect on the phosphorylation of mTOR (Fig. 8A–D). SBI-0206965 inhibited the phosphorylation of ULK1 and decreased the protein levels of LC3II/LC3I, Atrogin-1, and MuRF-1 in C2C12 myotubes but had no effect on the phosphorylation of AMPKα and mTOR (Fig. 8A–D). This suggests that AMPK, when phosphorylated, does not activate mTOR but instead directly activates ULK1, which stimulates autophagy. This shows that the AMPK/ULK1 pathway is indeed involved in the regulation of the autophagy and atrophy of C2C12 myotubes under hypoxia. In addition, iPSCs has the potential to protect against the autophagy and atrophy in C2C12 myotubes by inhibiting the AMPK/ULK1 pathway under hypoxic condition.

## Discussion

Skeletal muscle is a metabolically active tissue and a major protein store in the body. It has a certain tolerance to hypoxia, which disrupts the balance between muscle protein synthesis and degradation, leading to muscle atrophy [13]. Muscle atrophy is a complex multifactorial process that is not yet clearly understood. Under conditions of severe chronic hypoxia, the proliferation and differentiation of myoblasts are inhibited, and muscle regeneration is not possible, further exacerbating muscle atrophy [24, 25]. DMD is a progressive muscle atrophy disease caused by the absence of dystrophin, and hypoxia further promotes muscle atrophy, which may be one of the reasons for the progressive aggravation of DMD. In our study, C2C12 myotubes were exposed to a DG250 anaerobic workstation with oxygen deprivation for 24 h to simulate skeletal muscle hypoxia in DMD. We found that hypoxia increased the mRNA levels of Bax and decreased the mRNA levels of Bcl2 in C2C12 myoblasts. Chiel et al. found that hypoxia leads to a change in fiber size distribution and a lower average fiber cross-sectional area in male mice [26]. Similarly, some in vitro studies indicated a reduction in cell size and maximum skeletal muscle contractility when myotubes were exposed to hypoxic conditions [27, 28]. Our study reveals that hypoxia decreases the width of C2C12 myotubes, increases the expression of skeletal muscle atrophy-specific E3 ubiquitin ligases atrogin-1 and MuRF1, and increases the expression of LC3II/LC3I. Further, Rui et al. indicated that under hypoxic conditions, autophagy plays a negative role in muscle differentiation, and the inhibition of autophagy promotes cell survival and protects against myotube degradation [29]. However, Yang et al. showed that restoring autophagic flux protects skeletal muscles from oxidative stress in old C57BL/6 (14-month-old) mice [30]. Zhang et al. suggested that the myogenic differentiation of C2C12 myoblasts decreased under hypoxia for 7 days, which may be due to the inhibition of autophagy activity [31].

In our study, iPSCs exhibited reduced apoptosis and enhanced cellular activity. Gao et al. showed that iPSC-derived cardiac cell transplantation reduced apoptosis in the peri-scar border zone of swines [32]. Liang et al. found that iPSC-derived mesenchymal stem cells inhibit H_2_O_2_-induced mitochondrial fragmentation and apoptosis in human umbilical vein endothelial cells [33]. Skeletal muscle atrophy is accompanied by activation of the ubiquitin–proteasome and autophagy-lysosome systems. These two systems coordinate and participate in mediating the homeostasis of the muscle protein hydrolysis system [34]. Autophagy is widely recognized as a highly conserved homeostatic mechanism that degrades and recycles large amounts of cytoplasmic proteins and organelles through lysosomes. However, the current studies on the role of autophagy in skeletal muscle atrophy are inconclusive. In our study, autophagy and atrophy were simultaneously upregulated in C2C12 myotubes under hypoxic conditions. Some studies have suggested that inhibition of autophagy may be detrimental to skeletal muscles [35, 36]. However, autophagy activation has been shown to cause skeletal muscle atrophy [37, 38]. In our study, we found that the levels of atrophy upregulated in C2C12 myotubes were accompanied by the activation of autophagy and that the AMPK/mTOR/ULK1 autophagy-related pathway was also activated. Interestingly, the inhibition of the AMPK /ULK1 pathway simultaneously decreased autophagy and atrophy in C2C12 myotubes.

The results of our study demonstrate that the AMPK/ULK1 pathway, which is activated in C2C12 myotubes under hypoxic condition, is inhibited in C2C12 myotubes co-cultured with iPSCs. Thus, autophagy and atrophy of C2C12 myotubes may be ameliorated by iPSCs via downregulation of the AMPK/ULK1 pathway under hypoxic conditions. However, the exact underlying regulatory mechanisms remain unclear. It is possible that chronic hypoxia induces oxidative damage and AMPK phosphorylation in C2C12 myotubes, whereas iPSCs secrete cytokines against hypoxic damage by inhibiting AMPK under hypoxic conditions (Additional file 1: Figure S1). Polina et al. showed that the activation of AMPK phosphorylation not only restored the absence of dystrophin but also improved mitochondrial dysfunction and inhibited oxidative damage in the muscle progenitor cells of mdx mice [39]. Fang et al. found that a conditioned medium derived from hypoxia-stimulated adipose-derived stem cells promoted proliferation, migration, and tube formation by increasing CCL2, CCR2, TNF-α, TLR2, and TLR4 protein levels in human dermal microvascular endothelial cells [40]. An increasing number of studies have shown that stem cell transplantation repairs tissue damage in vivo, not only due to the regeneration of stem cells, but also due to the strong para-secretory function of stem cells, which can adapt to the changes of internal environment at any time. However, a limitation of the present study is the lack of in vivo experiments to verify the improvement in autophagy and atrophy after iPSC transplantation in a DMD animal model. In our study, the effectors secreted by iPSCs that activate the AMPK/ULK1 pathway to regulate autophagy and atrophy under hypoxic conditions still remain to be clarified. In addition, in vivo experiments should be conducted to confirm the effect of iPSCs transplantation on DMD hypoxic damage.

## Conclusion

In this study, we found that iPSCs enhanced the resistance of C2C12 myoblasts to hypoxia and inhibited apoptosis and autophagy in the presence of oxidative stress. Further, we showed that iPSCs improved the autophagy and atrophy of C2C12 myotubes and downregulated the phosphorylation of AMPKα and ULK1 in C2C12 myotubes, indicating that iPSCs protected C2C12 myotubes from hypoxia-induced autophagy and atrophy via the AMPK/ULK1 pathway. The results of our study have led us to propose a regulatory mechanism model for iPSCs in the treatment of hypoxic damage caused by muscular dystrophy, and provide a new theoretical basis for the treatment of muscular dystrophy in stem cells.

## Materials and methods

### C2C12 myoblast culture and differentiation

Mouse C2C12 myoblasts were purchased from iCell Bioscience, Inc. (China). The C2C12 myoblasts were cultured in Dulbecco’s Modified Eagle’s Medium (DMEM) high glucose (Gibco, USA) containing 10% fetal bovine serum (Gibco, USA) and 1% penicillin–streptomycin (Gibco, USA), and were differentiated into myotubes in DMEM high glucose containing 2% horse serum (Hyclone, USA) and 1% penicillin–streptomycin when the cell proliferation density of 80–90% in a 5% CO_2_ incubator at 37 °C. Medium was changed at least every 2 days.

### iPSCs culture and identification

The human iPSCs were obtained from Guangjin Pan Research Group, and were cultured in the mTeSR1 medium (STEMCELL, USA) at 37 °C in a 5% CO_2_ incubator. Digestion was performed with 0.5 mM EDTA solution, and cell culture plates were coated with Matrigel matrix (Corning, USA) before passaging. The iPSCs were then differentiated into three germ layers in Essential 6 medium (Gibco, USA) in low-adhesion dishes for seven days, with the medium changed every two days. On day eight of the suspension culture, spherical embryos were collected into 0.1% gelatin-coated cell culture dishes, and the embryos were exposed and cultured in Essential 6 medium for 7 days, with the medium changed every two days. The embryos were fixed and subjected to cellular immunofluorescence staining to detect the expression of endodermal, mesodermal, and ectodermal markers.

### Co-culture and hypoxic treatment

The iPSCs were cultured in mTeSR1 medium in Transwell inserts (SPL, Korea) coated with Matrigel matrix, and the cells were allowed to reach 70–80% confluence for co-culture. The C2C12 myoblasts were cultured with growth medium or differentiation medium in 6-well plates. The day before co-culture, the medium was exposed to a DG250 anaerobic workstation (Don Whitley Scientific, UK) containing a 5% CO_2_, 10% H_2_, and 85% N_2_ gas mixture for oxygen removal. The iPSCs were washed three times with phosphate buffered saline (PBS, BI, USA) and co-cultured with C2C12 myoblasts or C2C12 myotubes together in a Transwell inserts system (6-well plates) in a DG250 anaerobic workstation at 37 °C for 24 h of oxygen deprivation. Further, 1.5 mL of the medium was added to the Transwell insert and 2.5 mL of the medium was added to the 6-well plates.

### Calcein/PI cell viability and cytotoxicity assay

The activity of iPSCs was analyzed using the Calcein/PI Cell Viability/Cytotoxicity Assay Kit (Beyotime, China). When the iPSCs reached 60–70% confluence in six-well plates, they were placed in a DG250 anaerobic workstation at 37 °C for 24 h. After exposure to hypoxia for 24 h, iPSCs were washed once with PBS, and 1 mL of calcein AM/PI staining solution (1 uL/mL calcein acetoxymethyl ester and 1 uL/mL propidium iodide) was added. The iPSCs were then incubated at 37 °C for 30 min. After 24 h, iPSCs were observed under a fluorescence microscope (Leica Microsystems, Germany). Active cells were stained with calcein AM (green), and inactive cells were stained with PI (red).

### Mitochondrial membrane potential (MMP) assay

Analysis of C2C12 myoblast activity was performed using the Enhanced Mitochondrial Membrane Potential Assay Kit with JC-1 (JC-1, 5,5′,6,6′-Tetrachloro-1,1′,3,3′-tetraethyl-imidacarbocyanine iodide, molecular formula C_25_H_27_Cl_4_IN_4_) (Beyotime, China). After exposure to hypoxia for 24 h, the C2C12 myoblasts were washed once with PBS. Next, 1 mL of growth medium and 1 mL of JC-1 staining solution (5 uL/mL JC-1) were added, and the cells were incubated at 37 °C for 20 min. C2C12 myoblasts were then washed twice with PBS. Images were acquired using a fluorescence microscope (Leica Microsystems, Germany), and the relative proportions of red and green fluorescence were used as a measure of mitochondrial depolarization.

### Lactate dehydrogenase (LDH) release assay

The cell viability of C2C12 myoblasts was performed using the LDH Release Assay Kit (Beyotime, China). After exposure to hypoxia for 24 h, the supernatant of the C2C12 myoblast medium was collected and centrifuged at 400*g* for 5 min. Next, 120 μL of the supernatant medium was taken from each experiment group and 60 μL of LDH test solution (comprising 20 uL lactic acid solution, 20 uL INT (2-p-iodophenyl-3-nitrophenyl tetrazolium chloride) solution, and 20 uL diaphorase) was added to a new 96-well plate, which was incubated in a shaker at 25 °C for 30 min and protected from light. After 30 min, the absorbance of each well was measured at 450 nm using a microplate reader.

### Reactive oxygen species (ROS) assay

The hypoxic damage assay of the C2C12 myoblasts was performed using the Reactive Oxygen Species Assay Kit (Beyotime, China). After exposure to hypoxia for 24 h, the supernatant of the C2C12 myoblast medium was removed, 1 mL of serum-free medium with DCFH-DA (2',7'-dichlorodihydrofluorescein diacetate, 10 uM) was added, and the cells were incubated at 37 °C for 20 min. Finally, the C2C12 myoblasts were washed three times with PBS and observed under a fluorescence microscope (Leica Microsystems, Germany).

### 5-Ethynyl-20-deoxyuridine (EdU) assays

Cell proliferation was detected using the Cell-Light EdU Apollo® 567 In Vitro Kit (RIBOBIO, China). After exposure to hypoxia for 24 h, the C2C12 myoblasts medium supernatant was removed, 1 mL of growth medium with EdU (50 uM) was added to incubated at 37 °C for 2 h. After 2 h, cells were washed twice with PBS, and was fixed with 4% bparaformaldehyde in PBS for 30 min at 25 °C. After 30 min, cells were washed once with the glycine solution in PBS (2 mg/mL), treated with 0.5% TritonX-100 in PBS for 10 min and washed once with PBS. Next, C2C12 myoblasts were incubated with 500ul of 1× Apollo® 567 staining solution at 25 °C for 30 min protected from light. Finally, cells were washed twice with 0.5% TritonX-100 in PBS for 10 min each and were observed under a fluorescence microscope (Leica Microsystems, Germany).

### Flow cell cycle assay

Cell cycle was detected using Cell Cycle and Apoptosis Analysis Kit (Beyotime, China). After exposure to hypoxia for 24 h, C2C12 myoblasts were collected and centrifuged at 400×*g* for 5 min. The medium supernatant was removed, and cells were washed with 1 mL of pre-cooled PBS and transferred to a 1.5 mL centrifuge tube. Centrifuged as above, the supernatant was removed, 1 mL of pre-cooled 70% ethanol was added, and the cells were fixed at 4 °C for 30 min. The supernatant was removed, and the cells were washed with 1 mL of pre-cooled PBS and centrifuged again. Next, 0.5 mL of pre-prepared PI staining solution was added to each tube of cell samples, the cells were resuspended slowly, mixed well and incubated at 37 °C for 30 min protected from light. Flow cytometry was used to detect red fluorescence at an excitation wavelength of 488 nm, and cell DNA content analysis was performed using the specific software.

### Western blot

The separation gel and gel concentrate were prepared according to the instructions of the SDS-PAGE gel preparation kit (Beyotime, China). The total amount of protein sampled was 20 µg. For electrophoresis (Bio-Rad, USA), a constant voltage of 80 V was applied for 20–30 min, and a constant voltage of 120 V was applied for 60–70 min. For electrophoresis: PVDF membrane (0.45 um pore size, Millipore, USA), 260 mA constant current was applied for 90 min. For blocking: 5% skimmed milk powder (BD, USA) and shaker blocked at 25 °C for 1 h. Primary antibodies were incubated overnight at 4 °C. The next day, the membranes were incubated with secondary antibodies at 25 °C for 1 h. The target proteins were visualized using ECL chemiluminescence solution (Wanleibio, China). Strip grayscale values were analyzed using ImageJ software. The antibodies used are listed in Additional file 1: Table S1.

### qPCR

The RNA reverse transcription was prepared according to the instructions of the PrimeScript™ RT Master Mix (TaKaRa, Japan), and qPCR reaction programs was set up according to the instructions of TB Green® Premix Ex Taq™ II (TaKaRa, Japan). The obtained Cq values were used to calculate the relative expression of target gene mRNA based on the expression of 18S mRNA. The mRNA expression level of each sample was calculated using the 2^−∆∆CT^ method. The primers used are listed in Additional file 1: Table S2.

### Immunofluorescence

The cell medium supernatant was removed, and the C2C12 myoblasts were washed three times with PBS, fixed with methanol at − 20 °C for 15 min, treated with 0.3% TritonX-100 for 20 min, and blocked at 25 °C for 1 h. Primary antibodies were incubated overnight at 4 °C. Next day, conjugated fluorescent secondary antibodies were incubated at 25 °C in the dark for 1 h, and the DAPI staining solution (Beyotime, China) was incubated at 25 °C in the dark for 5 min. Images were acquired using a fluorescence microscope (Leica Microsystems, Germany). The antibodies used are listed in Additional file 1: Table S1.

### Myotube diameter measurement

Images were acquired using a fluorescence microscope, and the C2C12 myoblasts containing at least three nuclei were defined as myotubes. No less than 90 myotubes were sampled from each group. The short-axis diameter of the myotubes was measured using ImageJ software, and the maximum diameter of each myotube was defined as the myotube diameter.

### Statistical analysis

The experimental data are displayed as mean ± SEM, and each group of experiments was repeated at least three times. The original data were calculated and analyzed using GraphPadPrism8 analysis software. In this experiment, the differences in the means of multiple groups of data were compared using the analysis method of one-way ANOVA, and *P* < 0.05 was defined as statistically significant.

## Supplementary Information

**Additional file 1**: **Fig S1**, **Table S1**, **Table S2**. **Fig S1** illustrates the interrelationships between p-AMPK, p-mTOR, p-ULK1, autophagy, and atrophy. **Table S1** illustrates antibodies used in western blot and immunofluorescence assays in this study. **Table S2** illustrates designed primer sequences used for RT-PCR, where the mRNA sequence of all nine genes was obtained from the Gene database (www.ncbi.nlm.nih.gov), and primers were designed by a tool for finding specific primers, Primer-BLAST (https://www.ncbi.nlm.nih.gov/tools/primer-blast/).

## Data Availability

The datasets used during the current study are deposited and publicly available. Materials used in this study are commercially available.

## Abbreviations

DMD: Duchenne muscular dystrophy
iPSCs: Induced pluripotent stem cells
nNOS: Neuronal nitric oxide synthase
HIF: Hypoxia-induced factor
VEGF: Vascular endothelial growth factor
UPS: Ubiquitin–proteasome system
ALP: Autophagy-lysosome pathway
MuRF1: Muscle RING finger1
AMPK: AMP-activated protein kinase
ULK1: Unc-51 like in autophagy activating kinase 1
MSCs: Mesenchymal stem cells
EdU: 5-Ethynyl-20-deoxyuridine
