# Supplementary material for: iPSCs ameliorate hypoxia-induced autophagy and atrophy in C2C12 myotubes via the AMPK/ULK1 pathway
Source: Biol Res. 2023 Jun 3;56:29. doi: 10.1186/s40659-023-00435-4 (PMC10239182; doi:10.1186/s40659-023-00435-4)
Supplement: Supplementary file 1 — Additional file 1: Fig S1, Table S1, Table S2. Fig S1 illustrates the interrelationships between p-AMPK, p-mTOR, p-ULK1, autophagy, and atrophy. Table S1 illustrates antibodies used in western blot and immunofluorescence assays in this study. Table S2 illustrates designed primer sequences used for RT-PCR, where the mRNA sequence of all nine genes was obtained from the Gene database (www.ncbi.nlm.nih.gov), and primers were designed by a tool for finding specific primers, Primer-BLAST (https://www.ncbi.nlm.nih.gov/tools/primer-blast/). [file 40659_2023_435_MOESM1_ESM.docx]

**Supplementary Material**

**
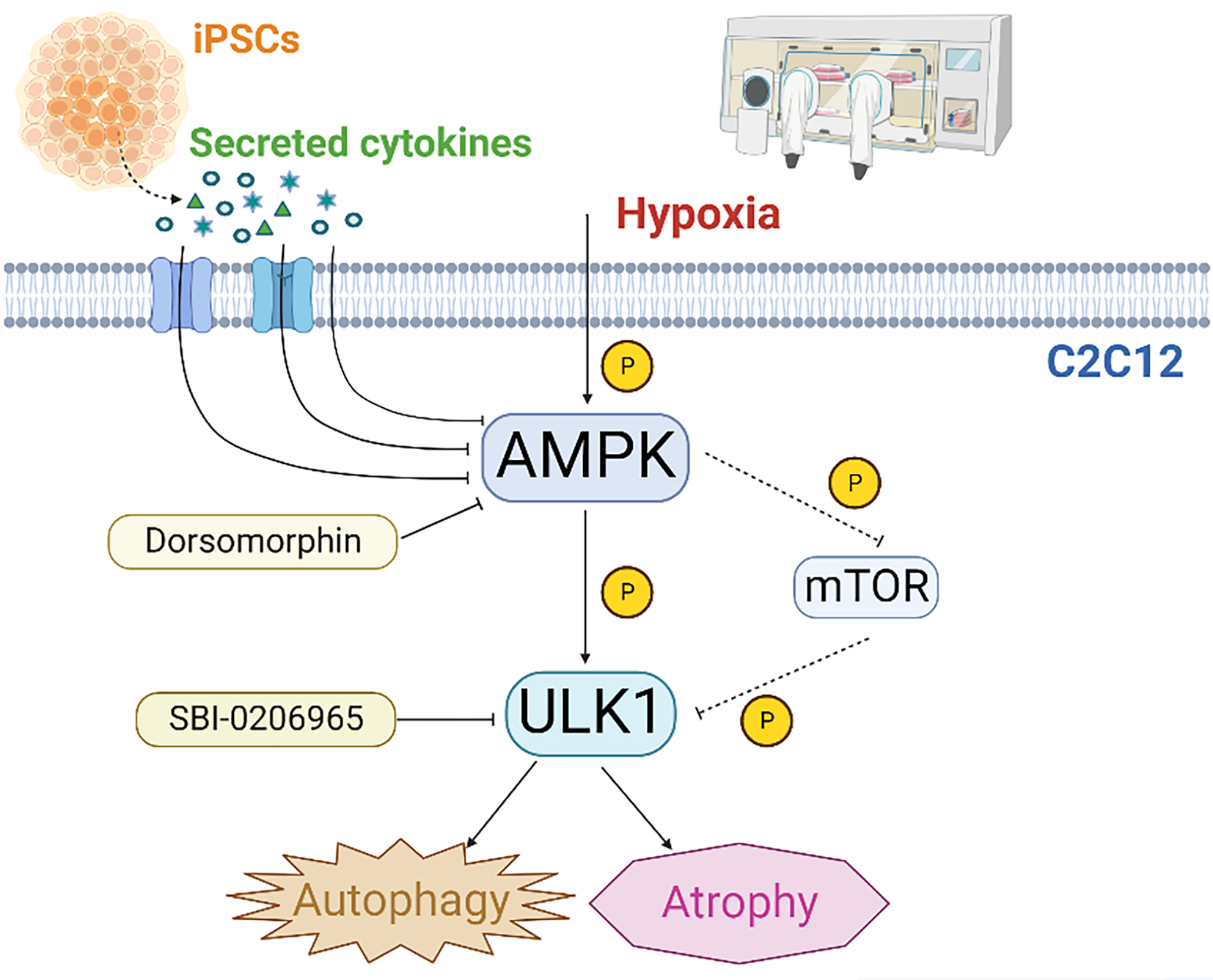
**

**Fig. S1 Schematic of the interrelationships between p-AMPK, p-mTOR, p-ULK1, autophagy, and atrophy.** Hypoxia stimulates phosphorylation of AMPK (p-AMPK), and p-AMPK induces phosphorylation of ULK1, which positively upregulates autophagy and atrophy. Induced pluripotent stem cells (iPSCs) may secrete cytokines to downregulate phosphorylation of AMPK to inhibit activation of autophagy and atrophy. In addition, p-AMPK can indirectly induce phosphorylation of the ULK1 by phosphorylating mTOR. Dorsomorphin is an AMPK inhibitor; SBI-0206965 is a ULK1 inhibitor. AMPK, 5' AMP-activated protein kinase; mTOR, mechanistic target of rapamycin; p, phosphorylated.

Table S1. Antibodies used in the study

| Primary Antibody | Clone | Company | Catalog No. | Dilution |
| --- | --- | --- | --- | --- |
| Bax | Polyclonal | Abcam | ab182733 | 1:1000 |
| SQSTM1 / p62 | Polyclonal | Abcam | ab109012 | 1:1000 |
| Fbx32 | Polyclonal | Abcam | ab168372 | 1:1000 |
| MURF1 + MURF3 + MURF2 | Polyclonal | Abcam | ab172479 | 1:1000 |
| LC3A/B (D3U4C) XP | Monoclonal | CST | 12741 | 1:1000 |
| Bcl-2 (D17C4) | Monoclonal | CST | 3498 | 1:1000 |
| AMPKα (D5A2) | Monoclonal | CST | 5831 | 1:1000 |
| Phospho-AMPKα (Thr172) (40H9) | Monoclonal | CST | 2535 | 1:1000 |
| mTOR (7C10) | Monoclonal | CST | 2983 | 1:1000 |
| Phospho-mTOR  (Ser2448)(D9C2) | Monoclonal | CST | 5536 | 1:1000 |
| ULK1 (D8H5) | Monoclonal | CST | 8054 | 1:1000 |
| Phospho-ULK1  (Ser555) (D1H4) | Monoclonal | CST | 5869 | 1:1000 |
| MyHC | Polyclonal | RD | MAB4470 | 1:100 |
| Oct-4A (C30A3) | Monoclonal | CST | 2840 | 1:200 |
| Peroxidase Affinipure | Polyclonal | YEASEN | 33101ES60 | 1:5000 |
| Sox2 (D6D9) | Monoclonal | CST | 3579 | 1:200 |
| SSEA4 (MC813) | Monoclonal | CST | 4755 | 1:200 |
| TRA-1-81 (TRA-1-81) | Monoclonal | CST | 4745 | 1:200 |
| FoxA2/HNF3β (D56D6) XP® | Monoclonal | CST | 8186 | 1:200 |
| Nestin | Polyclonal | Abcam | ab105389 | 1:00 |
| beta-Tubulin | Polyclonal | Affinity | T0023 | 1:10000 |
| Anti-Rabbit IgG  (H+L),F(ab`)2 Fragment(Alexa Fluor 488 Conjugate) | Polyclonal | CST | 4412 | 1:1000 |
| Anti-Mouse IgG (H+L), Polyclonal  F(ab`)2 Fragment  (Alexa Fluor 555 Conjugate) | Polyclonal | CST | 4409 | 1:1000 |

Table S2. Primer sequences for qPCR

| Genes | Primer Sequences（5' to 3'） |
| --- | --- |
| 18S | F:GTAACCCGTTGAACCCCATT R:CCATCCAATCGGTAGTAGCG |
| LC3A | F:CTGTCCTGGATAAGACCAAGTT R:GTCTTCATCCTTCTCCTGTTCA |
| LC3B | F:CCACCAAGATCCCAGTGATTAT R:TGATTATCTTGATGAGCTCGCT |
| P62 | F:GAACACAGCAAGCTCATCTTTC R:AAAGTGTCCATGTTTCAGCTTC |
| Bcl2 | F:GATGACTTCTCTCGTCGCTAC R:GAACTCAAAGAAGGCCACAATC |
| Bax | F:TTGCCCTCTTCTACTTTGCTAG R:CCATGATGGTTCTGATCAGCTC |
| MURF1 | F:CCAAGGAGAATAGCCACCAG R:CGCTCTTCTTCTCGTCCAG |
| Fbx32 | F:TTCACAAAGGAAGTACGAAGGA R:GCTGGTCTTCAAGAACTTTCAG |
| ATG12 | F:GCCTCGGAACAGTTGTTTATTT R:CAGTTTACCATCACTGCCAAAA |
